# Antagonists of growth hormone-releasing hormone suppress *in vivo* tumor growth and gene expression in triple negative breast cancers

**DOI:** 10.18632/oncotarget.634

**Published:** 2012-08-30

**Authors:** Roberto Perez, Andrew V. Schally, Irving Vidaurre, Ricardo Rincon, Norman L. Block, Ferenc G. Rick

**Affiliations:** ^1^ Veterans Affairs Medical Center, Miami, FL; ^2^ South Florida VA Foundation for Research and Education, Miami, FL; ^3^ Department of Pathology University of Miami, Miller School of Medicine, Miami, FL; ^4^ Divisions of Hematology/Oncology University of Miami, Miller School of Medicine, Miami, FL; ^5^ Endocrinology, Department of Medicine, University of Miami, Miller School of Medicine, Miami, FL

**Keywords:** neuropeptide analog, tumor gene expression, receptor silencing, targeted therapy, inflammation

## Abstract

This study evaluated the effects of a modern antagonistic analog of GHRH on tumor growth and on expression of inflammatory cytokine genes in two models of human triple negative breast cancers (TNBC). The TNBC subtype is refractory to the treatment options available for other hormone-independent breast cancers. Inflammatory cytokines play a major role in the cellular signaling associated with breast cancer pathogenesis and enhance epithelial-mesenchymal transitions (EMT), drug resistance, and metastatic potential. Growth hormone-releasing hormone (GHRH) is a hypothalamic neuropeptide which regulates the synthesis and release of growth hormone by the pituitary and is an autocrine/paracrine growth factor for multiple human cancers. The effects of analogs of GHRH on tumoral cytokine expression have not been previously investigated. Animals bearing xenografts of the human TNBC cell lines, HCC1806 and MX-1, were treated with MIA-602, an antagonistic analog of GHRH. Treatment with MIA-602 significantly reduced tumor growth. We quantified transcript levels of the genes for several inflammatory cytokines. Expression of INFγ, IL-1α, IL-4, IL-6, IL-8, IL-10, and TNFα, was significantly reduced by treatment with MIA-602. We conclude that treatment of TNBC with GHRH antagonists reduces tumor growth through an action mediated by tumoral GHRH receptors and produces a suppression of inflammatory cytokine signaling. Silencing of GHRH receptors *in vitro* with siRNA inhibited the expression of GHRH-R genes and inflammatory cytokine genes in HCC1806 and MX-1 cells. Further studies on GHRH antagonists may facilitate the development of new strategies for the treatment of resistant cancers.

## INTRODUCTION

Breast cancer is the leading cause of mortality in Hispanic and African-American women and the second most common cause of cancer-related death in Caucasian women. In the United States alone, nearly 200,000 women are afflicted with breast cancer each year and 41,000 die as a result of their malignancy.[1] These figures can be extrapolated to approximately 4 million new cases and 820,000 deaths per year, worldwide. Breast cancer is a very heterogeneous disease and encompasses several distinct entities. The subtype defined as triple negative breast cancer (TNBC) is negative for estrogen receptor, progesterone receptor, and the human epidermal growth factor receptor 2 (Her2). TNBC accounts for 10-15% of all breast cancer cases. This phenotype is hereditary, more invasive, affects younger women, and has a much poorer prognosis than other phenotypes.[2] Triple negative breast cancers are refractory to the treatment options available for other hormone-independent breast cancers, which are negative for estrogen receptor and progesterone receptor but positive for Her2. This accounts for the low survivability of TNBC.[3] Alternate treatment strategies must therefore be devised to address this clinical deficiency.

Inflammatory cytokines have been shown to play a major role in the cellular signaling involved in breast cancer pathogenesis.[4-6] Increased expression of inflammatory cytokines correlates with higher tumor grade and greater metastatic potential, both of which predict poorer survival. Inflammatory cytokines also enhance drug resistance in breast cancer.[7] Among the roles of cytokines in breast cancer is their ability to regulate epithelial-mesenchymal transitions (EMT).[8, 9] In the course of EMT, expression of intercellular adhesion molecules and other characteristics of an epithelial phenotype is lost, and cells acquire a stem-cell-like or “mesenchymal” phenotype. This phenotype is highly motile and possesses stem-cell-like properties including a high degree of resistance to chemotherapy and radiation. In addition to cancer stem-cell development and regulation of treatment resistance, EMT is the principal mechanism involved in metastasis and tumor invasion.[10-12] Disruption of the signaling pathways involved in EMT may therefore provide an effective treatment strategy for currently difficult to treat or untreatable cancers such as TNBC.

The potential use of neuropeptide analogs for the treatment of cancer has long been established.[13-15] Growth hormone-releasing hormone (GHRH) is a peptide hormone, secreted by the hypothalamus, which regulates the synthesis and release of growth hormone by the pituitary.[16, 17] Growth hormone subsequently stimulates the release of hepatic insulin-like growth factor, which is a major anabolic growth factor and a potent mitogen for many neoplasms.[17-20] Biologically active GHRH, mRNA for GHRH, GHRH receptors (GHRH-R), and GHRH-R splice variants have been identified in surgical specimens and tumor cell lines of a multitude of human cancers, including various types of breast cancer [21-28]. GHRH acts as an autocrine/paracrine growth factor in human cancers [16, 29-31] including breast [32]. Pituitary-type GHRH-R and splice variant 1 of GHRH-R appear to mediate the direct effects of GHRH and its analogs on tumors [33]. *In vitro* and *in vivo* proliferation of various human cancers is suppressed by antagonistic analogs of GHRH (referred to as “GHRH antagonists”) [19, 34-36]. These findings further support the concept of GHRH as a growth factor for clinical cancer.

*In vivo* studies have demonstrated the anti-tumor activity of GHRH antagonists against multiple cancer types [16, 29]. Studies of GHRH antagonists on prostate and lung cancers demonstrated the ability to modulate signaling pathways involved in cellular proliferation, survival, metastasis, and apoptosis [31, 37-39]. Among the affected pathways is the PI3K-AKT, which regulates inflammatory cytokines through NF-κβ.[37, 38] Treatment resistance in breast cancer is enhanced by activation of the NF-κβ pathway by inflammatory. [40, 41] *In vivo* studies of the effects of GHRH antagonists on benign prostatic hyperplasia, a partially inflammatory condition, resulted in significant decreases in prostate size after treatment [42]. Analyses indicate that treatment with GHRH antagonists suppresses the expression of pro-inflammatory cytokines in benign prostatic hyperplasia (BPH).[42, 43] These results also support the hypothesis that GHRH antagonists will suppress the expression of the inflammatory cytokines associated with breast cancer.

In this study, the human TNBC cell lines, HCC1806 and MX-1, were xenografted into nude mice to evaluate the effects of the GHRH antagonist MIA-602 on tumor growth and inflammatory cytokine gene expression. The animals were treated daily with subcutaneous injections of MIA-602 for five weeks, at which time tumors were collected for gene expression analysis. To confirm the effects of the GHRH antagonist on cytokine gene expression, cultures of HCC1806 and MX-1 were treated with small interfering RNA (siRNA) to silence the expression of GHRH-R genes. One-step real-time quantitative reverse transcription polymerase chain reaction (qRT-PCR) was used to analyze the expression of inflammatory cytokine genes.

## RESULTS

### Effect of GHRH Antagonist MIA-602 on the Growth of Xenografts of HCC1806 and MX-1 Human TNBC Breast Cancers

Treatment with the GHRH antagonist MIA-602 at a dosage of 5 μg/day was initiated after the tumors reached a volume of ~100 ± 7 mm^3^ and lasted for five weeks. Tumors that were treated with MIA-602 had significantly (*P* < 0.01) smaller volumes than controls after one week of treatment. Differences in volume were significant (*P* < 0.01) for the duration of the experiment.

**Figure 1:**
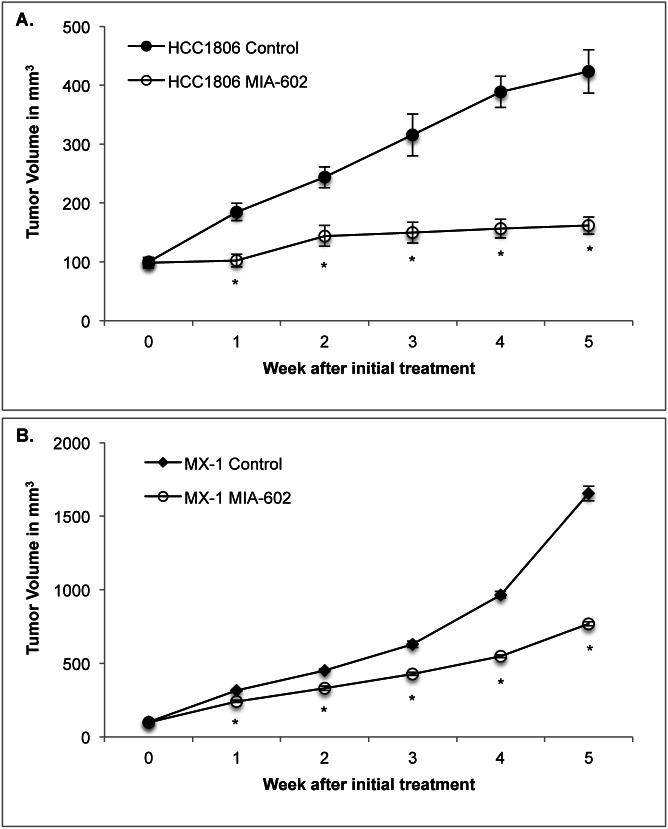
Treatment with the GHRH antagonist MIA-602 significantly reduces the growth of A HCC1806 and **B.** MX-1 human triple negative breast cancer tumors in nude mice. Animals in the experimental group were treated with subcutaneous administration of 5 μg of MIA-602 daily. Vertical bars indicate ± SEM, n=10 animals, * *P* < 0.01 vs. control

Treatment of HCC1806 tumors with MIA-602 significantly (*P* < 0.01) reduced mean tumor volume by 68% compared with control tumors. The mean HCC1806 tumor volume was 161.6 ± 14.6 mm^3^ for tumors treated with MIA-602 and 423.5 ± 37.1 mm^3^ for controls by the fifth week of the experiment (figure 1a).

Treatment of MX-1 tumors with MIA-602 also significantly (*P* < 0.01) decreased the mean tumor volume by 54% compared with control tumors. The mean MX-1 tumor volume was 769.1 ± 14.6 mm^3^ for tumors treated with MIA-602 and 1654.5 ± 49.8 mm^3^ for controls by the fifth week of the experiment (figure 1b).

### Expression of GHRH and GHRH-R mRNA by HCC1806 and MX-1 Human TNBC Breast Tumors

Protein and mRNA for GHRH and GHRH-R were found in both HCC1806 and MX-1 human TNBC cell lines. Expression of tumoral GHRH and GHRH-R mRNA was determined after five weeks of treatment using qRT-PCR.

**Figure 2:**
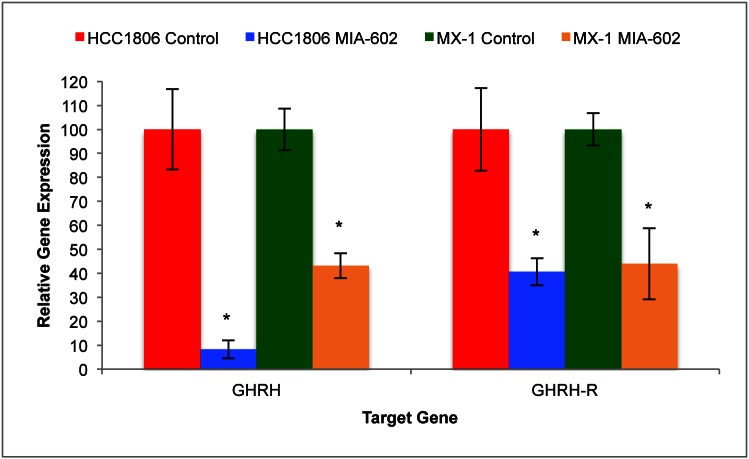
Treatment of tumors with the GHRH antagonist MIA-602 significantly suppressed the expression of GHRH and GHRH-R genes by tumors of HCC1806 and MX-1 human TNBC Vertical bars indicate ± SEM, n=12 tumors, * *P* < 0.01 vs. control.

Expression of GHRH and GHRH-R genes by HCC1806 human TNBC tumors was significantly (*P* < 0.05) suppressed by treatment with MIA-602. HCC1806 tumors treated with the GHRH antagonist for five weeks expressed 91.8% (±3.8%) less mRNA for GHRH and 59.4% (±5.7%) less mRNA for GHRH-R than controls (figure 2a). Expression of GHRH and GHRH-R genes by MX-1 human TNBC tumors was also significantly (*P* < 0.05) suppressed by treatment with MIA-602. MX-1 tumors treated with the GHRH antagonist for five weeks expressed 56.2% (±5.2%) less mRNA for GHRH and 56.1% (±14.9%) less GHRH-R mRNA than controls (figure 2b).

### Expression of mRNA for Proinflammatory IFNγ, IL-1α, IL-4, IL-6, IL-8, IL-10, and TNFα Genes by HCC1806 and MX-1 Human TNBC Breast Tumors

**Figure 3:**
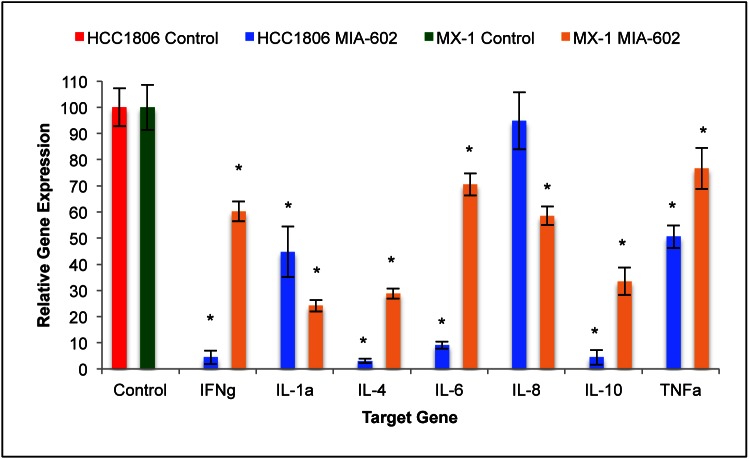
Treatment of tumors with the GHRH antagonist MIA-602 suppressed the expression of IFNγ, IL-1α, IL-4, IL-6, IL-8, IL-10, and TNFα Treatment of HCC1806 human TNBC resulted in significant suppression of all of the analyzed genes except IL-8. Treatment of MX-1 human TNBC resulted in significant suppression of all of the analyzed genes. Vertical bars indicate ± SEM, n=12 tumors, * *P* < 0.01 vs. control.

Treatment with the GHRH antagonist, MIA-602, suppressed the expression of proinflammatory cytokine genes by both HCC1806 and MX-1 human TNBC. Expression levels of interferon gamma (IFNγ) mRNA and tumor necrosis factor alpha (TNFα) mRNA by MIA-602 treated HCC1806 tumors were 95.6% (±2.6%) and 49.4% (±4.4%) less than the control group, respectively. Expression of interleukin 1 alpha (IL-1α), IL-4, IL-6, IL-8, and IL-10 was 55.3% (±9.6%), 97.0% (±0.9%), 91.0% (±1.3%), 5.2% (±10.9%), and 95.6% (±2.8%) less than control, respectively (figure 3a). Treatment of HCC1806 tumors with the GHRH antagonist resulted in significant (*P* < 0.01) reductions in the expression of mRNA for all genes analyzed with the exception of that for interleukin 8 (IL-8).

Expression levels of IFNγ mRNA and TNFα mRNA by MIA-602 treated MX-1 tumors was 39.8% (±3.8%) and 23.3% (±7.9%) less than the control group, respectively. Expression of mRNA for IL-1α, IL-4, IL-6, IL-8, and IL-10 was 75.8% (±2.2%), 71.2% (±1.9%), 29.4% (±4.3%), 47.5% (±3.5%), and 66.5% (±5.2%) less than control, respectively (figure 3b). Treatment of MX-1 tumors with the GHRH antagonist resulted in significant (*P* < 0.01) reductions in the expression of mRNA for all genes analyzed.

### Effect of GHRH-R Gene Silencing on GHRH mRNA and GHRH-R mRNA Expression by HCC1806 and MX-1 Human TNBC Cell Lines

**Figure 4:**
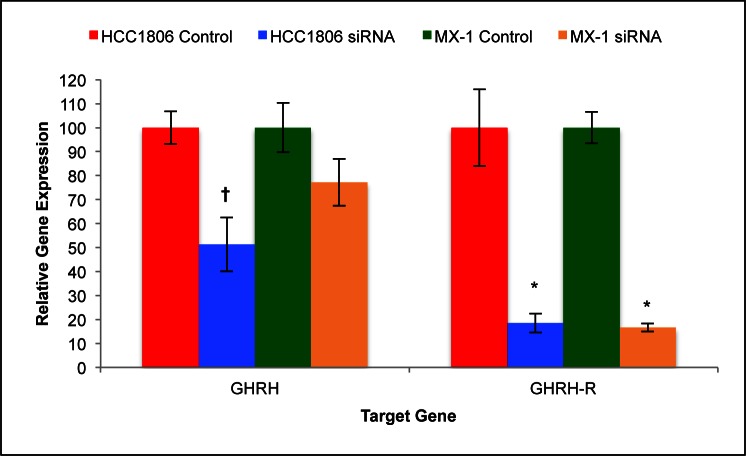
Silencing of GHRH-R mRNA with siRNA significantly suppressed GHRH gene expression by HCC1806 but not by MX-1 Silencing resulted in an 80% reduction in the expression of GHRH-R genes by both HCC1806 and MX-1 cells. Vertical bars indicate ± SEM, n=9 samples, * *P* < 0.01 vs. control, † *P* < 0.05 vs. control.

Expression levels of mRNA for GHRH and GHRH-R was determined after silencing the expression of GHRH-R genes with siRNA and culturing the cells for seven days. Expression of GHRH mRNA and GHRH-R mRNA by HCC1806 human TNBC cells was significantly (*P* < 0.01) suppressed by silencing of GHRH-R genes with siRNA. Cells of HCC1806 expressed 48.7% (±11.2%) less GHRH mRNA and 81.9% (±4.0%) less GHRH-R mRNA than controls (figure 4a). This analysis indicates that the silencing of GHRH-R genes with siRNA was 82% (±4%) efficient.

Expression of GHRH mRNA by MX-1 human TNBC cells was not significantly suppressed by silencing of GHRH-R with siRNA. Contrarily, expression of GHRH-R mRNA by MX-1 human TNBC cells was significantly (*P* < 0.01) suppressed. Cells of MX-1 expressed 32.8% (±9.8%) less GHRH mRNA and 83.4% (±1.7%) less GHRH-R mRNA than controls (figure 4b). This analysis indicates that silencing of GHRH-R genes in MX-1 human TNBC cells was 83% (±2%) efficient.

### Effect of GHRH-R Gene Silencing on Proinflammatory Cytokine Gene Expression by HCC1806 and MX-1 Human TNBC Cell Lines

**Figure 5:**
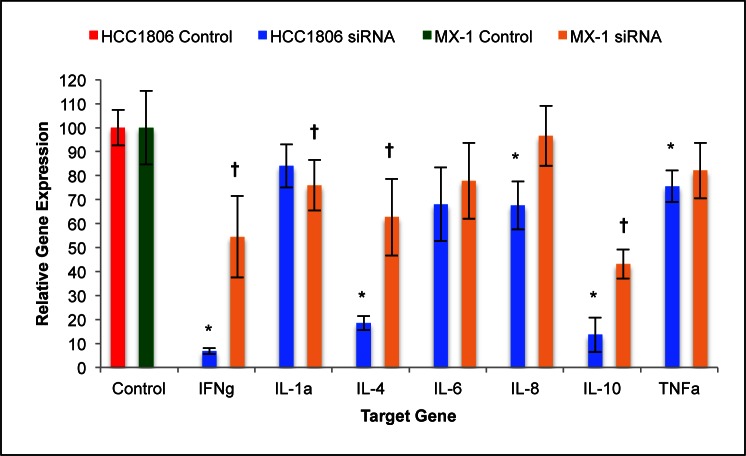
Silencing of GHRH-R expression by human TNBC cell lines with siRNA suppressed the expression of IFNγ, IL-1α, IL-4, IL-6, IL-8, IL-10, and TNFα Silencing of HCC-1806 human TNBC cells resulted in significant suppression of all of the analyzed genes except IL-1α, IL-6, and TNFα. Silencing of MX-1 human TNBC cells resulted in significant suppression of all of the analyzed genes except IL-6, IL-8, and TNFα. Vertical bars indicate ± SEM, n=9 samples, * *P* < 0.01 vs. control, † *P* < 0.05 vs. control.

Silencing of GHRH-R genes with siRNA suppressed the expression of proinflammatory cytokine mRNA by cells of HCC1806 and MX-1 human TNBC. Expression levels of IFNγ mRNA and TNFα mRNA by silenced cells of HCC1806 were 93.2% (±1.2%) and 24.5% (±6.6%) less than in the control group, respectively. Expression of mRNA for IL-1α, IL-4, IL-6, IL-8, and IL-10 genes was 16.0% (±9.0%), 81.5% (±2.9%), 32.0% (±15.4%), 32.4% (±10.0%), and 86.4% (±7.2%) lower than in control, respectively (figure 5a). Silencing of HCC1806 cells with GHRH-R siRNA resulted in significant (*P* < 0.01) reductions in the expression of mRNA for all genes analyzed with the exception of TNF-α, IL-1α, and IL-6.

Expression levels of IFNγ mRNA and TNFα mRNA by silenced cells of MX-1 were 45.5% (±16.9%) and 17.9% (±11.6%) smaller than the in control group, respectively. Expression of mRNA for IL-1α, IL-4, IL-6, IL-8, and IL-10 genes was 24.1% (±10.5%), 37.4% (±16.0%), 22.2% (±15.8%), 3.4% (±12.5%), and 66.9% (±6.0%) lower than in control, respectively (figure 5b). Silencing of MX-1 cells with GHRH-R siRNA resulted in significant (*P* < 0.05) reductions in the expression of mRNA for all genes analyzed with the exception of TNFα, IL-6, and IL-8.

## DISCUSSION

Much information has now been accumulated concerning the role of GHRH, GHRH receptors, and receptor splice variants (SV) in carcinogenesis. Proliferation of some human cancers *in vitro* is stimulated by exogenous GHRH and suppressed by GHRH antagonists or GHRH anti-sera.[44] Studies suggest that dysregulation of GHRH expression or autocrine/paracrine secretion contributes to the pathogenesis of breast and other cancers.[45] *In vivo* studies have demonstrated the anti-tumor activity of GHRH antagonists against multiple cancer types. Studies of GHRH antagonists on prostate and lung cancers demonstrated their ability to modulate signaling pathways involved in cellular proliferation, survival, metastasis, and apoptosis. Among the affected pathways is the PI3K-AKT pathway which regulates the expression of inflammatory cytokines and the initiation of EMT.[37, 38]

Several reports have detailed the effects of treatment with GHRH antagonists on the growth of breast cancers.[16, 28, 46, 47] However, the role of GHRH in the regulation of inflammatory cytokine expression in triple negative breast cancer has not been fully investigated. This study illustrates the benefits of using GHRH antagonists for the treatment of cancers which are highly treatment-resistant.

Inflammation is a key regulatory process in breast cancer progression and severity. Inflammatory cytokines are seldom or minimally expressed in the normal breast epithelia, but they are significantly elevated in several breast cancer subtypes.[48] In addition, relapse and metastasis are associated with significantly increased and prolonged expression of TNFα, IL-1, and IL-6 indicating progression-related roles for these cytokines.[8, 48] Other studies have demonstrated that prolonged exposure of breast tumor cells to inflammatory cytokines leads to EMT, which is the principle mechanism associated with generation of cancer stem-cells, development of treatment resistance, and the initiation and progression of metastasis.[7, 8, 11, 12]

Numerous studies have firmly established the regulatory role of inflammatory cytokines in cancer.[2, 4-8, 11, 48] We recently reported the reduction of prostate size and suppression of inflammatory cytokines by GHRH antagonists in an experimental BPH rodent model.[42] This study supplements our previous work by demonstrating similar effects on *in vivo* cytokine gene expression in HCC1806 and MX-1 human TNBC. Analysis of the mRNA levels of the genes expressed by tumors treated with MIA-602 indicates suppressed expression of tumoral inflammatory cytokines.

These results suggest that GHRH antagonists can be used to inhibit the generation of cancer stem cells, treatment resistance, and metastatic potential by suppressing the expression of inflammatory cytokine genes. Given these conclusions, GHRH antagonists may provide effective treatment of patients suffering from difficult to treat or treatment resistant cancers such as TNBC.

## MATERIALS AND METHODS

### Ethics Statement

Investigation has been conducted in accordance with the ethical standards and according to the Declaration of Helsinki and according to national and international guidelines and has been approved by the authors’ institutional animal care and usage committee (Chairman Dr. Carlos Perez-Stable, Miami VA Medical Center).

### Drugs and Chemicals

The GHRH antagonistic analog, MIA-602, was synthesized in our laboratory as previously described.[18, 49] MIA-602 was dissolved in a 0.1% / 10% DMSO/propylene glycol solution for daily subcutaneous injection.

### Animals

Female nude mice (Harlan Laboratories) between 10 and 11 weeks of age (20g body weight) were housed in a climate-controlled environment with a 12-h light/dark cycle and were fed standard laboratory diet with water *ad libitum*. Body weights were determined weekly. All animals remained healthy throughout the experiment. Animal care was in accordance with institutional guidelines and complied with National Institutes of Health policy.

### Cell Culture

Cultures of the human triple negative breast cancer cell line, HCC1806, were maintained in RPMI 1640 medium supplemented with 10% FBS. Cultures were keep in a humidified incubator in a 5% CO_2_ atmosphere at 37^°^C. Growth medium was replaced every 72 hours for two weeks. Cells were collected using 0.05% trypsin and incubating at 37^°^C for 3 minutes. Trypsin was inactivated with an equal volume of FBS containing medium and the cells were collected by centrifugation at 1000 × g for 10 minutes.

### Study Design

Donor animals were injected subcutaneously with 10^5^ cells and tumors were allowed to grow for 4 weeks. The tumors were collected postmortem and cut into approximately 5 mg fragments. Fragments were rinsed with sterile PBS and xenografted subcutaneously into both flanks of each study animal. Tumors were allowed to grow to a mean volume of ~100 mm^3^ prior to initiation of treatment. Animals were randomly assigned to one of two experimental groups. The control group remained untreated and the treated group received daily subcutaneous injection of MIA-602 (5μg/day) for 5 weeks. Tumor volume and body weight were assessed weekly. All animals were euthanized by cervical dislocation upon study completion. The tumors were collected postmortem and immediately submerged in RNAlater stabilization solution (Ambion).

### Small Interfering RNA Gene Silencing

Silencing of GHRH-R was accomplished by reverse transfection using the siPORT NeoFX Transfection Reagent and Silencer Select siRNA (Applied Biosystems). Cells were trypsinized immediately before silencing. Cell suspensions were centrifuged at 3000 × g for 10 minutes and the media removed. Cells were suspended to a density of 10^5^ cells/ml in fresh media containing 10% FBS and antibiotic. RNA (1μM) was diluted 1:4 in opti-MEM and 100μl of this solution combined with 100μl of a 1:10 NeoFX solution for each well. Transfection complexes were allowed to form for 15 minutes at room temperature. In each well of a 6 well culture plate, 2ml of cell suspension was combined with 200μl of complexes and cultured at 37^°^C and 5% CO_2_ for 7 days, replacing the medium and transfection complexes once.

### RNA Isolation

Excised tumors were immediately cut into 25 mg pieces and submerged in RNAlater stabilization solution. After an overnight incubation at 4^°^C, for thorough stabilization, samples were homogenized in lysis buffer, and total RNA was isolated using the GE Illustra RNAspin Isolation Kit (GE Healthcare) according to the manufacturer's protocol. Contaminating DNA was eliminated with an on-column DNase treatment as part of the isolation procedure. Total RNA was quantified and assessed for purity using a Nanodrop spectrophotometer (Thermo Scientific).

### SYBR Green-based RT-PCR Primer Design

Gene expression was determined using qRT-PCR. All RNA targets were analyzed using custom designed oligonucleotide primers designed for use in SYBR green based qRT-PCR. The assays were painstakingly designed using extremely strict parameters in order to exclude non-human (mouse) templates and target regions of low energy secondary structures, maximizing both specificity and sensitivity. All assays were determined to produce a single product which was verified as the human target of interest by DNA sequencing.

Transcript specific primers were designed using the Beacon Designer software suite (Premiere Biosoft) with modified parameters. Primer searches were limited to regions on mRNA sequences (Refseqs) which were not homologous to the equivalent mRNA from mice (*Mus musculus*). The resulting human-specific sequences were screened for regions of stable secondary structures (ΔG < -3.0 Kcal/mol), which were excluded from our primer search. Primer searches were optimized for reverse transcription at 52^°^C and fast cycling PCR with single step annealing/extension at 57^°^C. Primer hairpin energy was limited to ΔG = -3.0 Kcal/mol and dimer energies were limited to ΔG = -4.0 Kcal/mol. Dimers including the last 3 bases of the 3' end of the primer were limited to ΔG = -2.0 Kcal/mol. Primers were designed to result in amplicons of 75-200 bp in length. Primer pairs that were less than 98% efficient were excluded. All primers used produce a single product of predictable and reproducible melting temperature (Tm). All primers were optimized and verified by sequencing the corresponding amplicons.

### Real-time Quantitative Reverse-Transcription Polymerase Chain Reaction (qRT-PCR)

Gene expression analysis was conducted using one-step qRT-PCR with SYBR green chemistry. This method conducts the reverse transcription reaction and PCR in a single tube format from 20ng total RNA template. The production of the PCR amplified gene product is monitored using the fluorescence resulting from the binding of SYBR green to the double stranded DNA amplicons. Reactions were conducted in a CFX96 Real-Time System using the One-Step SYBR Green qRT-PCR reaction kit (Bio-Rad). Reactions were conducted in triplicate and normalized to three internal standard genes using the δδCt method.[50]

### Statistical Analysis

Prism 5 software (Graphpad Software, Inc.) was used for statistical evaluation of the data. Results are expressed as means ± SEM. One-way ANOVA followed by Bonferroni *t* test or a two-tailed Student's *t* test was used where appropriate, and significance was accepted at *P* < 0.05.
